# Prevalence and factors associated with digital addiction among students taking university entrance tests: a GIS-based study

**DOI:** 10.1186/s12888-024-05737-9

**Published:** 2024-04-25

**Authors:** Firoj Al-Mamun, Md Emran Hasan, Nahida Bintee Mostofa, Marzia Akther, Tahnin Mashruba, Mohammad Arif, Amatul Haque Chaahat, Anha Bushra Salam, Maksuda Akter, Md Al Asif Abedin, Md. Imtiaj Ahmad Bulbul, Md Shoeb Adnan, Md. Shafiul Islam, Mst. Shauda Ahmed, Md. Sultan Mahmud Shahin, Saiful Islam, Mumtaz Mohammed Hussain, Abdullah Al Habib, Moneerah Mohammad ALmerab, David Gozal, Mohammad Muhit, Nitai Roy, Mohammed A. Mamun

**Affiliations:** 1 CHINTA Research Bangladesh, Savar, Dhaka, 1342 Bangladesh; 2 Department of Public Health and Informatics, Jahangirnagar University, Savar, Dhaka, Bangladesh; 3 Department of Public Health, University of South Asia, Dhaka, Bangladesh; 4 Software College, Northeastern University, Shenyang, China; 5 Department of Geography and Environment, Jahangirnagar University, Savar, Dhaka, Bangladesh; 6 Department of Ayurvedic Medicine, Institute of Teaching & Research in Ayurveda, Jamnagar, Gujarat India; 7 Dental Unit, Sir Salimullah Medical College, Mitford, Dhaka Bangladesh; 8 Department of Pharmacy, Jahangirnagar University, Savar, Dhaka, Bangladesh; 9 Department of Marketing, Jahangirnagar University, Savar, Dhaka, Bangladesh; 10 Dental Unit, Sher-E-Bangla Medical College, Barisal, Bangladesh; 11 Department of Biochemistry and Molecular Biology, University of Rajshahi, Rajshahi, Bangladesh; 12 Department of Audiology and Speech Language Pathology, Bangladesh University of Professionals, Dhaka, Bangladesh; 13 Department of Agriculture, Bangladesh Agricultural University, Mymensingh, Bangladesh; 14 Department of Biotechnology and Genetic Engineering, Jahangirnagar University, Savar, Dhaka, Bangladesh; 15 Department of Public Administration, Begum Rokeya University, Rangpur, Bangladesh; 16 Department of Public Health, North South University, Dhaka, Bangladesh; 17 Department of Government and Politics, Jahangirnagar University, Savar, Dhaka Bangladesh; 18 Department of Psychology, College of Education and Human Development, Princess Nourah bint Abdulrahman University, Riyadh, Saudi Arabia; 19 Joan C. Edwards School of Medicine, Marshall University, 25701, Huntington, WV USA; 20 Department of Biochemistry and Food Analysis, Patuakhali Science and Technology University, Patuakhali, Bangladesh

**Keywords:** Digital addiction, Social media addiction, Mental health, Digital media, Students

## Abstract

**Background:**

The surge in digital media consumption, coupled with the ensuing consequences of digital addiction, has witnessed a rapid increase, particularly after the initiation of the COVID-19 pandemic. Despite some studies exploring specific technological addictions, such as internet or social media addiction, in Bangladesh, there is a noticeable gap in research focusing on digital addiction in a broader context. Thus, this study aims to investigate digital addiction among students taking the university entrance test, examining its prevalence, contributing factors, and geographical distribution using GIS techniques.

**Methods:**

Data from a cross-sectional survey were collected from a total of 2,157 students who were taking the university entrance test at Jahangirnagar University, Bangladesh. A convenience sampling method was applied for data collection using a structured questionnaire. Statistical analyses were performed with SPSS 25 Version and AMOS 23 Version, whereas ArcGIS 10.8 Version was used for the geographical distribution of digital addiction.

**Results:**

The prevalence of digital addiction was 33.1% (mean score: 16.05 ± 5.58). Those students who are attempting the test for a second time were more likely to be addicted (42.7% vs. 39.1%), but the difference was not statistically significant. Besides, the potential factors predicted for digital addiction were student status, satisfaction with previous mock tests, average monthly expenditure during the admission test preparation, and depression. No significant difference was found between digital addiction and districts. However, digital addiction was higher in the districts of Manikganj, Rajbari, Shariatpur, and Chittagong Hill Tract areas, including Rangamati, and Bandarban.

**Conclusions:**

The study emphasizes the pressing need for collaborative efforts involving educational policymakers, institutions, and parents to address the growing digital addiction among university-bound students. The recommendations focus on promoting alternative activities, enhancing digital literacy, and imposing restrictions on digital device use, which are crucial steps toward fostering a healthier digital environment and balanced relationship with technology for students.

**Supplementary Information:**

The online version contains supplementary material available at 10.1186/s12888-024-05737-9.

## Introduction

Digital addiction refers to the addictive behaviors toward those technological devices that are electronic or digital. There are different types of digital addiction, for instance, smartphone addiction, internet addiction, social media addiction, etc. A recent systematic review and meta-analysis of 495 studies including 2,123,762 participants from 64 countries reported that the pooled prevalence of digital addiction ranged between 6.04 and 26.99% [1]. Having said that people with excessive and uncontrolled use of digital media and devices are more likely to report suicidal behaviors. For instance, a US nationally representative study of data from 2009 to 2017 belonging to the project, Youth Risk Behavior Surveillance System, conducted within a total of 72,942 youths, found that a higher score on digital media use frequency increased the greater risk of suicidal behavior [2]. Another study on Chinese adolescents found recurrent self-harm to be at 1.86 and 1.45 times higher for those who used the internet for ≥ 2 h and 2–3 h on weekdays, respectively [3].

After the inception of the COVID-19 pandemic, online engagement has increased dramatically, and addictive behaviors towards digital media have reportedly increased. For instance, a meta-analysis by Marciano et al. [4] explored the role of digital media use on mental health problems. The magnitude of digital addiction has increased during the pandemic as per evidence from meta-analysis [1], and social media use and media addiction were associated with a higher degree of mental health suffering [4]. People in Bangladesh like other countries, were more engaged in online communication during the pandemic, resulting in more time spent with digital gadgets and media. For instance, more than half of students (53.2%) reported using the internet more than 5 h daily [5], which is reportedly higher than the previous study conducted within a similar group before the pandemic (20.7%) [6]. More engagement with internet increases the risk of addictive behaviors and problematic use of digital gadgets and media related to internet, as observed during the pandemic in the country [5].

In Bangladesh, a number of studies have been carried out assessing different types of digital addiction among different student populations. In most of the studies assessing problematic internet or smartphone use, university students have been prioritized for consideration as the study population [7–9], whereas a few studies were conducted among adolescent students [10, 11]. Of the studies among adolescent students, Islam et al. [10] provide insight into internet addiction and loneliness during the COVID-19 pandemic, where their prevalence rates were found to be 88.25% and 72.51% respectively. Another study conducted afterward found a significant relationship between gadget addiction and cognitive function, suggesting that digital media addiction can negatively impact cognitive performance [11]. However, no studies have ever been conducted among students transitioning from high school to university.

Evidently, the transition to university is one of the crucial periods for adolescents, and the previous studies observed substantial mental health problems in this group. For instance, nearly half of students appearing university entrance test in Bangladesh, had suffered from mental health problems, depression (47.9%), anxiety (28.9%), and burnout (43.7%), and the risk of suicidality was observed at 4.12, 3.48, and 1.71 times, respectively, higher than those who are not with those problems [12]. This is not a surprising finding as Bangladeshi students have to pass a very competitive entrance exam for the public university, whereas around 55,000 seats are available for nearly a million high school graduates [13, 14], and the nature of different syllabuses aligning with different faculties or institutes (e.g., medical college, agricultural universities, etc.), also make them vulnerable to suffering from psychological stress and burnout related symptoms [12, 15]. Previously, it has been reported that burnout mediates the relationship between interpersonal stress and internet addiction [16].

This study aims to bridge existing gaps by investigating digital addiction among students transitioning from high school to university, a demographic largely unexplored in the prior studies. Unlike previous studies that focused on specific type of technological addiction, such as internet or social media addiction, this study delves into more general aspect of digital addiction. With respect to the nationwide distribution of technological addiction, no studies provide GIS-based insights, which was done in this study along with gender-based and student status-based spatial mappings. Importantly, the findings of this study not only promise a deeper understanding of digital addiction dynamics but also offer valuable guidance for targeted interventions and policy initiatives. The findings of this study are anticipated being helpful to mitigate the adverse effects of digital addiction, contributing to the creation of a healthier digital ecosystem for the youth population in Bangladesh. Through the application of GIS, this study can identify areas with higher prevalence rates, facilitating precise interventions to address the magnitude of the digital addiction problem.

## Methods

### Study design, participants and procedure

In this cross-sectional study, the study participants were those students who were taking the university entrance test at Jahangirnagar University, Dhaka, Bangladesh. The entrance test was held between June 18 to 25, 2023, and data collection took place within this time frame. The participants deemed eligible for inclusion in this study were those individuals partaking in the university entrance test while concurrently residing in the university dormitories during the stipulated entrance test period. Students were approached for participating in this study at night who resided in the dormitories. The selection of the participants was facilitated through a convenience sampling approach, wherein all students present during the data collection phase were approached for solicit their participation. A noteworthy response was garnered from a total of 2,533 students who actively contributed to the study by responding to the survey. A key facet of the recruitment process involved a strategic briefing session conducted by the research team prior to participants’ active involvement. This session served the purpose of acquainting participants with the intricate terms and concepts embedded within the survey questionnaire, as well as on the study’s aims and objectives, potential benefits, and risks. However, after eliminating incomplete questionnaires, data from 2,157 participants were kept for final analysis.

### Measures

#### Sociodemographic factors

Sociodemographic information, including variables such as gender, permanent residence (rural and urban), religion, family type, and monthly family income. Participant’s family was categorized into three distinct groups: those with incomes less than 15,000 Bangladeshi Taka (BDT), those falling within the 15,000–30,000 BDT range, and those with monthly family incomes exceeding 30,000 BDT as following the previous study within the similar population [17]. For religion, others represent those studies belonging to a religion other than Muslim, that is, Hindu, Christianity, Buddhism, etc.

#### Admission-related variables

In Bangladesh, it is a common practice for most universities to permit students to attempt the entrance test at most twice. Therefore, data regarding the participants’ test-taking status were collected, distinguishing whether they were first-time test takers or had appeared on the test for a second time. Additionally, information related to their educational background in high school (Science, Commerce, and Arts), and GPA of their previous public exams at high schools were collected. Participants were queried about whether they had sought guidance from professionals or coaching centers during their test preparation, as well as, their satisfaction on mock tests was recorded. Their average monthly expenditures related to test preparation and the specific type of university to which they aspired for admission were also asked.

#### Digital device-related variables

Digital device-related information is primarily divided into two key categories: the utilization of digital media platforms and the assessment of screen time. In particular, participants were asked if they used a number of digital devices or gadgets including TV, PC, Smartphone, and Gaming device. Whereas, the screen time of different digital activities was collected and categorized as per a previous study conducted by Garmy et al. [18]. And following this study, students were classified into two clear-cut groups: those who exceeded 2 h of daily digital device usage and those who maintained a usage duration of less than or equal to 2 h per day.

#### Patient health questionnaire

The Patient Health Questionnaire (PHQ-9) was used to evaluate levels of depression [19]. The PHQ-9 comprises of nine distinct items, each requiring respondents to indicate their experiences over the past two weeks using a four-point Likert scale (0 = not at all, 1 = several days, 2 = more than half of the days, and 3 = nearly every day). The cumulative scale ranges from 0 to 27, with higher scores signifying greater severity of depression [19]. A score equal to or more than 10 is typically indicative of depression. For this study, Cronbach’s alpha coefficient, which measures internal consistency, demonstrated a good level of reliability at 0.83.

#### Generalized anxiety disorder

The Generalized Anxiety Disorder (GAD-7) was used to evaluate levels of anxiety [20]. The GAD-7 comprises a total of seven items, with participants responding to them using a four-point Likert scale (0 = not at all, 1 = several days, 2 = more than half of the days, and 3 = nearly every day), reflecting their experiences over the past two weeks. The overall scale ranges from 0 to 21, and higher scores are indicative of more severe anxiety. Typically, a score equal to or more than 10 is considered an indicator of anxiety [20]. For this study, Cronbach’s alpha coefficient, which measures internal consistency, demonstrated an excellent level of reliability at 0.90.

#### Digital addiction scale

The Digital Addiction Scale (DAS) was developed using a modified version of Bergen Facebook Addiction Scale [21]. In this study, “Facebook” was replaced with digital devices and/or gadgets for each of the items, e.g., “*Used digital device in order to forget about personal problems?*”. For this modification, we performed Exploratory Factor Analysis (EFA), and Confirmatory Factor Analysis (CFA). The KMO measure of sampling adequacy was 0.845 (*p* < 0.001). In the EFA, the six items have explained a 48.68% variance. In CFA, the goodness of fit indices generated excellent values (χ^2^/df = 5.89, RMSEA = 0.04 (90% CI [0.03, 0.06]), SRMR = 0.02, CFI = 0.98, NFI = 0.98, GFI = 0.99]. Both EFA and CFA results suggest that the scale is valid and reliable in assessing digital addiction among university students. The items are responded to on a 5-point Likert scale from 1 (very rarely) to 5 (very often). The total score ranges from 6 to 30. The cutoff point for the scale was determined using the ROC curve. The Area Under Curve had a value of 1, indicating a perfect classifier at a cutoff point of 18.5. The sensitivity for this cutoff score was 81.8% and specificity was 100% [22]. For this study, Cronbach’s alpha coefficient demonstrated a good level of reliability at 0.79. The scale items are presented in the Supplementary material.

### Statistical analysis

Data collection and entry procedures were conducted using Google Forms, following which the collected data were formatted for the final analysis utilizing SPSS 25 and AMOS 23 software. The analysis consisted of the application of both descriptive and inferential statistics, with all analyses being conducted for the entire sample and subgroups categorized by the student status (first-time test takers vs. repeat test takers). Descriptive statistics were calculated, utilizing frequencies and percentages, mean and SD, skewness, and kurtosis. Normality assumptions were checked using skewness (<|3|) and kurtosis (<|10|) values of the variables [23]. Besides, Kolmogorov-Smirnov test was performed to check the normal distribution of the data. Both the EFA and CFA were performed to validate the scale. In the CFA, we used the criteria suggested by Hu and Bentler, where RMSEA < 0.05, SRMR < 0.05, GFI > 0.95, CFI > 0.95, and NFI > 0.95 indicated excellent model fit [24]. Furthermore, mean comparisons, including independent sample t-tests and one-way ANOVA tests analyzed within the data. Linear regression was also employed using the backward selection method to identify the potential predictors of digital addiction. Besides, a spatial analysis of digital addiction was conducted using ArcGIS 10.8 software. For this purpose, geographic data was sourced from https://www.diva-gis.org/. Initially, data from the total participants were aggregated according to districts. Subsequently, *post-hoc* analyses were performed and the outcomes were visualized using the maps, stratifying the results by gender and student status. In all statistical tests, a significance level of 0.05 was adopted.

### Ethics statement

This study was conducted in full compliance with the principles outlined in the Helsinki Declaration of 1975, with its subsequent revisions in 2008. Besides, all steps involving human participants and patients received formal approval from the review board of CHINTA Research Bangladesh [ref: chinta/2023/5]. Prior to enrolling participants in this study, they were provided with a briefing regarding the study’s objectives and purposes. Importantly, they were made fully aware of their right to decline participation or withdraw from the study at any point, thus emphasizing the paramount importance of informed and voluntary consent. This study participation required informed written consent from the participants.

## Results

### Characteristics of the participants

Table 1 presents the characteristics of the participants (*N* = 2,157; 53.5% females). About 67.7% belonged to rural areas, 78.6% were from nuclear families, and 34.3% were from middle-income families. Regarding admission-related variables, 61.9% were first-time test takers, 76% were coached by a professional or coaching center, 32.1% satisfied with their mock tests, and 49.6% had middle average monthly expenditure. In terms of digital device-related information, 19.4% of the participants reported that they watch TV, 12.4% used PC, 97% had a smartphone and 2.9% played games; whereas 93.4%, 74%, 87.8%, and 70.8% reported that they used screen time ≤ 2 hours daily for TV, gaming, chat, and video, respectively. Besides, 57.2% had reported suffering from depression, and 43.8% had anxiety (Table 1). In terms of student status, those students who were repeat test takers exhibited a non-significantly higher prevalence of digital addiction than first-time test takers (42.7% vs. 39.1%; χ^2^ = 2.736, *p* = 0.098).

**Table 1:** Characteristics of the participants

Variable	Total sample, n (%)	First-time test takers, n (%)	Repeat test takers, n (%)	χ^2^ value	p-value
Sociodemographic variables					
**Gender**					
Male	1004 (46.5)	591 (44.3)	413 (50.2)	7.297	**0.007**
Female	1153 (53.5)	744 (55.7)	409 (49.8)		
**Permanent residence**					
Rural	1460 (67.7)	881 (66.0)	579 (70.4)	3.891	0.049
Urban	668 (31.0)	433 (32.4)	235 (28.6)		
**Religion**					
Muslim	1838 (85.2)	1124 (84.2)	714 (86.9)	2.828	0.093
Others	302 (14.0)	200 (15.0)	102 (12.4)		
**Family type**					
Nuclear	1696 (78.6)	1059 (79.3)	637 (77.5)	1.506	0.220
Joint	419 (19.4)	248 (18.6)	171 (20.8)		
**Monthly income (BDT)**					
Low Income (< 15,000)	405 (18.8)	221 (16.6)	184 (22.4)	11.604	**0.003**
Middle income (15,000–30,000)	739 (34.3)	453 (33.9)	286 (34.8)		
High income (> 30,000)	536 (24.8)	351 (26.3)	185 (22.5)		
**Admission-related variables**					
**Secondary school certificate GPA**					
Poor (< 4.5)	314 (14.6)	136 (10.2)	178 (21.7)	82.851	**< 0.001**
Moderate (4.5–4.99)	609 (28.2)	344 (25.8)	265 (32.2)		
High (5)	1159 (53.7)	807 (60.4)	352 (42.8)		
**Higher secondary school certificate GPA**					
Poor (< 4.5)	125 (5.8)	85 (6.4)	40 (4.9)	2.430	0.297
Moderate (4.5–4.99)	534 (24.8)	323 (24.2)	211 (25.7)		
High (5)	1420 (65.8)	876 (65.6)	544 (66.2)		
**Coached by professional or coaching center**					
Yes	1639 (76.0)	1165 (87.3)	474 (57.7)	248.797	**< 0.001**
No	481 (22.3)	151 (11.3)	330 (40.1)		
**Desired institute/department for admission**					
Varsity	1457 (67.5)	877 (65.7)	580 (70.6)	18.002	**< 0.001**
Medical	514 (23.8)	346 (25.9)	168 (20.4)		
Engineering	123 (5.7)	89 (6.7)	34 (4.1)		
Agriculture	10 (0.5)	3 (0.2)	7 (0.9)		
**Satisfied with previous mock tests**					
Yes	690 (32.1)	440 (33.0)	250 (30.4)	0.003	0.958
No	1271 (58.9)	812 (60.8)	459 (55.8)		
**Average monthly expenditure on test preparation (BDT)**					
Low Expenditure (< 5000)	300 (13.9)	151 (11.3)	149 (18.1)	36.716	**< 0.001**
Middle Expenditure (5000–10,000)	1069 (49.6)	682 (51.1)	387 (47.1)		
High expenditure (> 10,000)	310 (14.4)	229 (17.2)	81 (9.9)		
**Educational background**					
Science	1530 (70.9)	1005 (75.3)	525 (63.9)	35.012	**< 0.001**
Arts	559 (25.9)	288 (21.6)	271 (33.0)		
Commerce	59 (2.7)	38 (2.8)	21 (2.6)		
**Digital device-related variables**					
***Digital medium used***					
**TV**					
Yes	418 (19.4)	269 (20.1)	149 (18.1)	1.333	0.248
No	1739 (80.6)	1066 (79.9)	673 (81.9)		
**PC**					
Yes	267 (12.4)	166 (12.4)	101 (12.3)	0.010	0.920
No	1890 (87.6)	1169 (87.6)	721 (87.7)		
**Smartphone**					
Yes	2093 (97.0)	1298 (97.2)	795 (96.7)	0.465	0.495
No	64 (3.0)	37 (2.8)	27 (3.3)		
**Gaming**					
Yes	63 (2.9)	40 (3.0)	23 (2.8)	0.070	0.791
No	2094 (97.1)	1295 (97.0)	799 (97.2)		
***Screen Time***					
**TV**					
>2 h/day	22 (6.6)	12 (5.6)	10 (8.5)	1.101	0.294
≤2 h/day	311 (93.4)	204 (94.4)	107 (91.5)		
**Gamming**					
>2 h/day	77 (26.0)	57 (28.6)	20 (20.6)	2.182	0.140
≤2 h/day	219 (74.0)	142 (71.4)	77 (79.4)		
**Chat**					
>2 h/day	95 (12.6)	59 (12.6)	36 (12.6)	0.000	0.992
≤2 h/day	658 (87.8)	409 (87.4)	249 (87.4)		
**Video**					
>2 h/day	278 (29.2)	191 (31.8)	87 (24.8)	5.243	**0.022**
≤2 h/day	674 (70.8)	410 (68.2)	264 (75.2)		
**Mental Health Problems**					
**Depression**					
No	628 (29.1)	396 (29.7)	232 (28.2)	0.317	0.574
Yes	1233 (57.2)	761 (57.0)	472 (57.4)		
**Anxiety**					
No	1067 (49.5)	670 (50.2)	397 (48.3)	1.299	0.254
Yes	945 (43.8)	570 (42.7)	375 (45.6)		

BDT, Bangladeshi Taka; GPA, Grade Point Average, TV, Television; PC, Personal Computer

### Item level analysis of the Bangla digital addiction scale

**Fig. 1:**
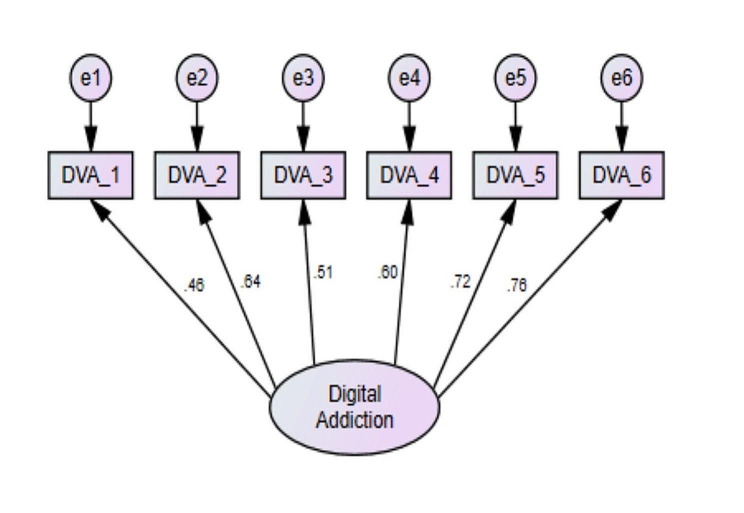
Confirmatory factor analysis of digital addiction scale

All the items had a skewness and kurtosis value in the range of ± 2, indicating a normal distribution. Factor loadings were greater than 0.32, indicating an acceptable fit [25]. The CFA has been presented in Fig. 1. Corrected item-total correlations were > 0.40, indicating all the items correlated with the total. The Cronbach’s alpha if an item were deleted was at a satisfactory level, therefore no item needs to be deleted [26]. The overall Cronbach’s alpha for the scale is 0.79 (Table 2).

**Table 2:** Item level analysis of the Bangla digital addiction scale

DAS item	Mean ± SD	Skewness	Kurtosis	Factor loadings (CFA)	Corrected item-total correlation	Cronbach’s alpha if item deleted
Item 1	2.23 ± 1.23	0.42	-0.74	0.46	0.41	0.78
Item 2	2.87 ± 1.28	-0.09	-0.92	0.64	0.57	0.74
Item 3	2.83 ± 1.36	-0.05	-1.12	0.51	0.45	0.77
Item 4	2.70 ± 1.42	0.11	-1.05	0.60	0.52	0.75
Item 5	2.48 ± 1.33	0.3	-0.93	0.72	0.60	0.73
Item 6	2.94 ± 1.37	-0.16	-1.10	0.76	0.64	0.72

### Pearson correlation among digital addiction and depression, anxiety

Table 3 reported the Pearson correlation between digital addiction and depression, and anxiety. The digital addiction scale was significantly correlated with depression (*r* = 0.48, *p <* 0.01) and anxiety (*r* = 0.42, *p <* 0.01), showing strong convergent validity **(**Table 3**)**.

**Table 3:** Pearson correlation among digital addiction and depression, anxiety

Variables	Mean ± SD	1	2	3
Digital Addiction (1)	16.05 ± 5.57	1		
Depression (2)	12.42 ± 5.84	0.48**	1	
Anxiety (3)	9.53 ± 5.65	0.42**	0.71**	1

**Correlation is significant at the 0.01 level (2-tailed)

### Mean differences in digital addiction

Table 4 presents the mean differences in digital addiction. The overall mean score for digital addiction was 16.06 ± 5.58 (out of 30). Male gender had a significantly higher mean score of digital addiction in three samples (for the total sample, t = 2.617, *p* < 0.001; for first-time test takers, t = 1.978, *p* < 0.001, and repeat test takers, t = 1.610, *p* = 0.012). However, students belonging to rural areas had a significantly higher mean score of digital addiction than urban ones, for the only total sample (t = 0.515, *p* = 0.037). In addition, students suffering from mental health problems were found to have higher scores of digital addictions than those without mental health suffering (for example, 17.56 ± 5.42 vs. 13.07 ± 4.66, *p* < 0.001, for depression; and 18.30 ± 5.47 vs. 14.16 ± 4.93, *p* = 0.010, for anxiety) (Table 4).

**Table 4:** Mean differences in digital addiction

Variables	Total sample			First-time test takers			Repeat test takers		
	Mean & SD	F/t	p-value	Mean ± SD	F/t	p-value	Mean ± SD	F/t	p-value
**Sociodemographic variables**									
**Gender**									
Male	16.40 ± 6.01	2.617	**< 0.001**	16.28 ± 5.97	1.978	**< 0.001**	16.56 ± 6.08	1.610	**0.012**
Female	15.76 ± 5.15			15.67 ± 5.08			15.92 ± 5.30		
**Permanent residence**									
Rural	16.10 ± 5.69	0.515	**0.037**	16.08 ± 5.64	1.145	0.059	16.14 ± 5.78	-0.719	0.400
Urban	15.97 ± 5.34			15.71 ± 5.24			16.46 ± 5.51		
**Religion**									
Muslim	16.03 ± 5.62	-0.341	0.133	15.90 ± 5.56	-0.535	0.167	16.22 ± 5.71	0.077	0.546
Other	16.15 ± 5.29			16.13 ± 5.17			16.18 ± 5.56		
**Family Type**									
Nuclear	15.95 ± 5.61	-1.833	0.514	15.88 ± 5.51	-0.985	0.812	16.07 ± 5.77	-1.622	0.157
Joint	16.51 ± 5.52			16.26 ± 5.51			16.87 ± 5.52		
**Monthly income (BDT)**									
Low Income	16.09 ± 5.97	0.289	0.749	15.91 ± 6.00	0.311	0.733	16.29 ± 5.94	0.033	0.967
Middle income	15.90 ± 5.50			15.72 ± 5.48			16.19 ± 5.53		
High Income	16.12 ± 5.45			16.02 ± 5.33			16.31 ± 5.69		
**Admission-related variables**									
**Secondary school certificate GPA**									
Poor (< 4.5)	16.10 ± 5.99	0.326	0.722	16.62 ± 6.08	1.630	0.196	15.70 ± 5.92	0.783	0.457
Moderate (4.5–4.99)	16.14 ± 5.64			16.06 ± 5.65			16.24 ± 5.64		
High (5)	15.93 ± 5.37			15.75 ± 5.24			16.34 ± 5.64		
**Higher secondary school certificate GPA**									
Poor (< 4.5)	16.30 ± 5.44	0.187	0.829	16.59 ± 5.51	0.732	0.481	15.68 ± 5.29	0.174	0.840
Moderate (4.5–4.99)	15.96 ± 5.77			15.78 ± 5.60			16.22 ± 6.03		
High (5)	16.03 ± 5.55			15.91 ± 5.40			16.22 ± 5.60		
**Coached by professional or coaching center**									
Yes	16.11 ± 5.54	0.849	0.531	15.95 ± 5.48	0.401	0.487	16.50 ± 5.70	1.436	0.758
No	15.86 ± 5.68			15.76 ± 5.59			15.91 ± 5.74		
**Desired institute/department for admission**									
Varsity	16.10 ± 5.72	0.558	0.643	16.01 ± 5.65	1.280	0.280	16.23 ± 5.82	0.397	0.755
Medical	15.86 ± 5.37			15.66 ± 5.28			16.26 ± 5.53		
Engineering	16.44 ± 4.99			16.12 ± 4.94			17.26 ± 5.13		
Agriculture	17.10 ± 6.70			21.00 ± 3.61			15.43 ± 7.21		
**Satisfied with previous mock tests**									
Yes	15.88 ± 5.53	-1.097	0.583	15.89 ± 5.35	-0.201	0.228	15.86 ± 5.84	-1.531	0.400
No	16.17 ± 5.63			15.95 ± 5.61			16.55 ± 5.64		
**Average monthly expenditure on test preparation (BDT)**									
<5000	15.98 ± 5.87	0.547	0.579	15.46 ± 5.67	1.081	0.340	16.51 ± 6.04	1.081	0.516
5000–10,000	15.99 ± 5.46			16.00 ± 5.41			15.96 ± 5.55		
>10,000	16.35 ± 5.52			16.31 ± 5.53			16.48 ± 4.97		
**Educational background**									
Science	15.92 ± 5.44	2.398	0.091	15.78 ± 5.34	2.272	0.104	16.19 ± 5.64	0.272	0.762
Arts	16.34 ± 5.91			16.33 ± 6.00			16.35 ± 5.82		
Commerce	17.19 ± 5.89			17.26 ± 5.26			17.05 ± 6.01		
**Digital device-related variables**									
**Digital medium used**									
**TV**									
Yes	16.12 ± 5.48	0.254	0.805	15.90 ± 5.37	-0.150	0.634	16.52 ± 5.66	0.652	0.864
No	16.04 ± 5.60			15.96 ± 5.53			16.18 ± 5.72		
**PC**									
Yes	15.89 ± 5.73	-0.531	0.605	15.78 ± 6.04	-0.386	**0.043**	16.07 ± 5.21	-0.322	0.080
No	16.08 ± 5.56			15.97 ± 5.42			16.26 ± 5.78		
**Phone**									
Yes	16.08 ± 5.57	0.834	0.190	15.94 ± 5.49	-0.093	0.534	16.29 ± 5.69	1.390	0.311
No	15.48 ± 5.93			15.03 ± 5.74			14.74 ± 6.21		
**Gaming**									
Yes	16.46 ± 5.58	0.582	0.987	15.58 ± 5.24	-0.432	0.789	18.00 ± 5.93	1.501	0.957
No	16.05 ± 5.58			15.96 ± 5.51			16.19 ± 5.70		
**Screen time**									
**TV**									
>2 h/day	15.91 ± 5.22	0.083	0.476	16.67 ± 5.52	0.858	0.607	15.00 ± 4.97	-1.064	0.749
≤2 h/day	15.81 ± 5.63			15.23 ± 5.64			16.91 ± 5.46		
**Gamming**									
>2 h/day	16.60 ± 5.86	0.090	0.764	16.31 ± 6.18	-0.107	0.664	17.70 ± 4.81	0.596	0.617
≤2 h/day	16.53 ± 5.62			16.31 ± 5.84			16.94 ± 5.18		
**Chat**									
>2 h/day	16.15 ± 5.41	-0.153	0.420	16.41 ± 4.98	0.330	0.157	15.72 ± 6.09	-0.656	0.619
≤2 h/day	16.24 ± 5.75			16.15 ± 5.74			16.40 ± 5.77		
**Video**									
>2 h/day	16.18 ± 5.32	0.117	0.405	16.10 ± 5.22	0.694	0.391	16.33 ± 5.60	-0.501	0.954
≤2 h/day	16.13 ± 5.57			15.78 ± 5.50			16.68 ± 5.64		
**Mental health problems**									
**Depression**									
No	13.07 ± 4.66	-18.602	**< 0.001**	12.93 ± 4.48	-15.458	**0.002**	13.31 ± 4.94	-10.479	0.053
Yes	17.56 ± 5.42			17.51 ± 5.31			17.65 ± 5.60		
**Anxiety**									
No	14.16 ± 4.93	-17.736	**0.010**	14.06 ± 4.82	-14.209	**0.020**	14.34 ± 5.13	-10.486	0.238
Yes	18.30 ± 5.47			18.26 ± 5.42			18.37 ± 5.56		

BDT, Bangladeshi Taka; GPA, Grade Point Average, TV, Television; PC, Personal Computer

### Predictive model for digital addiction

Table 5 presents the final predictive models for digital addiction. A multiple regression analysis utilizing a backward selection approach was employed herein. The final model was significant (F = 11.552, *p* < 0.001) identifying student status (*p* = 0.029), satisfaction with previous mock tests (*p* = 0.004), average monthly expenditure (*p* = 0.024), and depression (*p* < 0.001) as the potential predictive factors. The model explained a 56.2% variance in predicting digital addiction (Table 5).

**Table 5:** Final predictive model for digital addiction

Variables	R^2^ = 0.562, F = 11.552, *p* < 0.001; DW value = 1.924				
	B	S.E.	β	t	p
(Constant)	-6.210	3.825		-1.624	**0.113**
Student Status ^a^	2.957	1.300	0.258	2.274	**0.029**
Satisfied with previous mock tests ^b^	4.515	1.473	0.346	3.066	**0.004**
Average monthly expenditure (BDT)	0.000217	0.000092	0.271	2.363	**0.024**
Depression	0.566	0.099	0.635	5.732	**< 0.001**

B: unstandardized regression coefficient; β: standardized regression coefficient; ^a^1 = First time test taker, 2 = Repeat test taker; ^b^1 = Yes, 2 = No.

### Digital addiction across districts

**Fig. 2:**
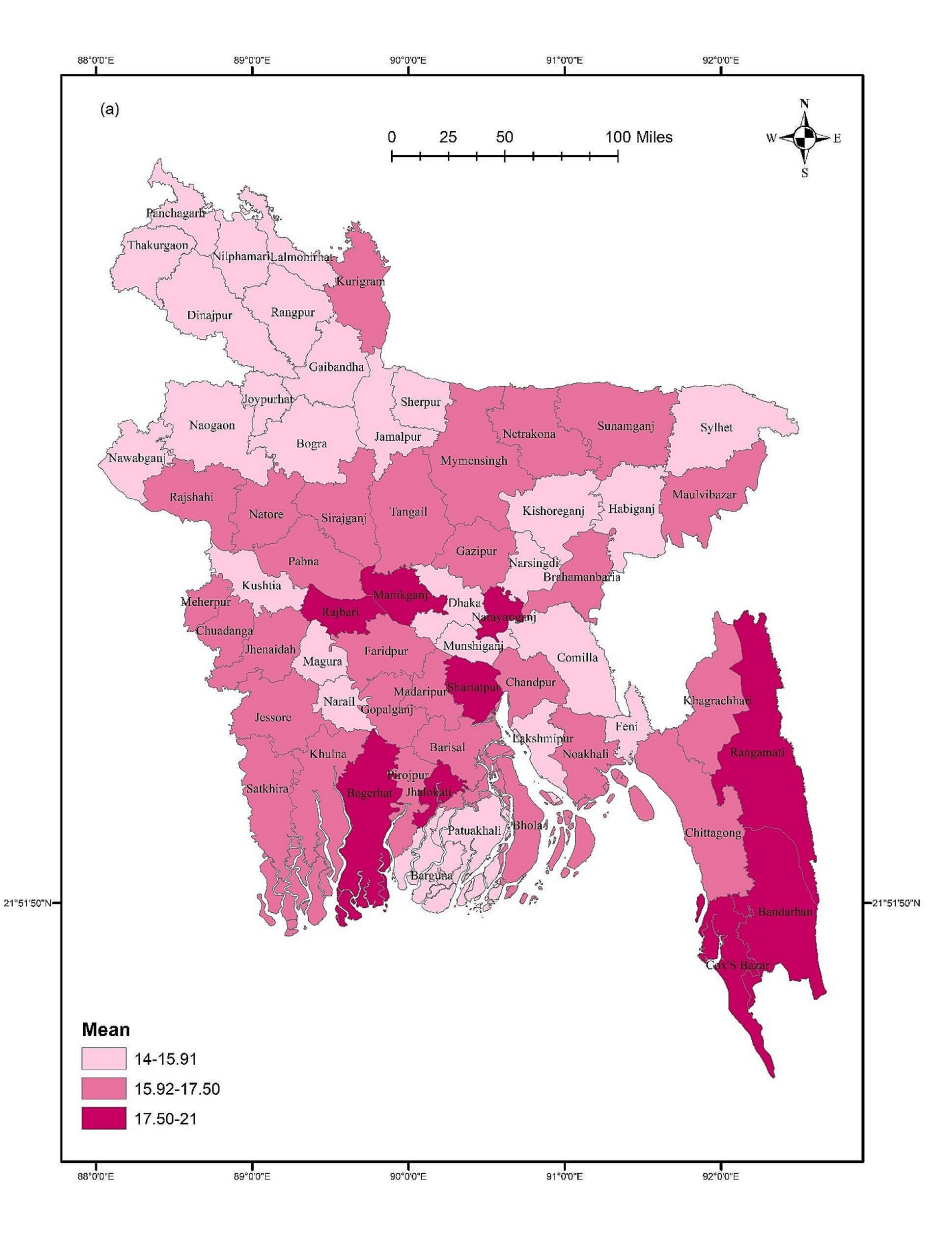
Mean score of digital addiction across districts

**Fig. 3:**
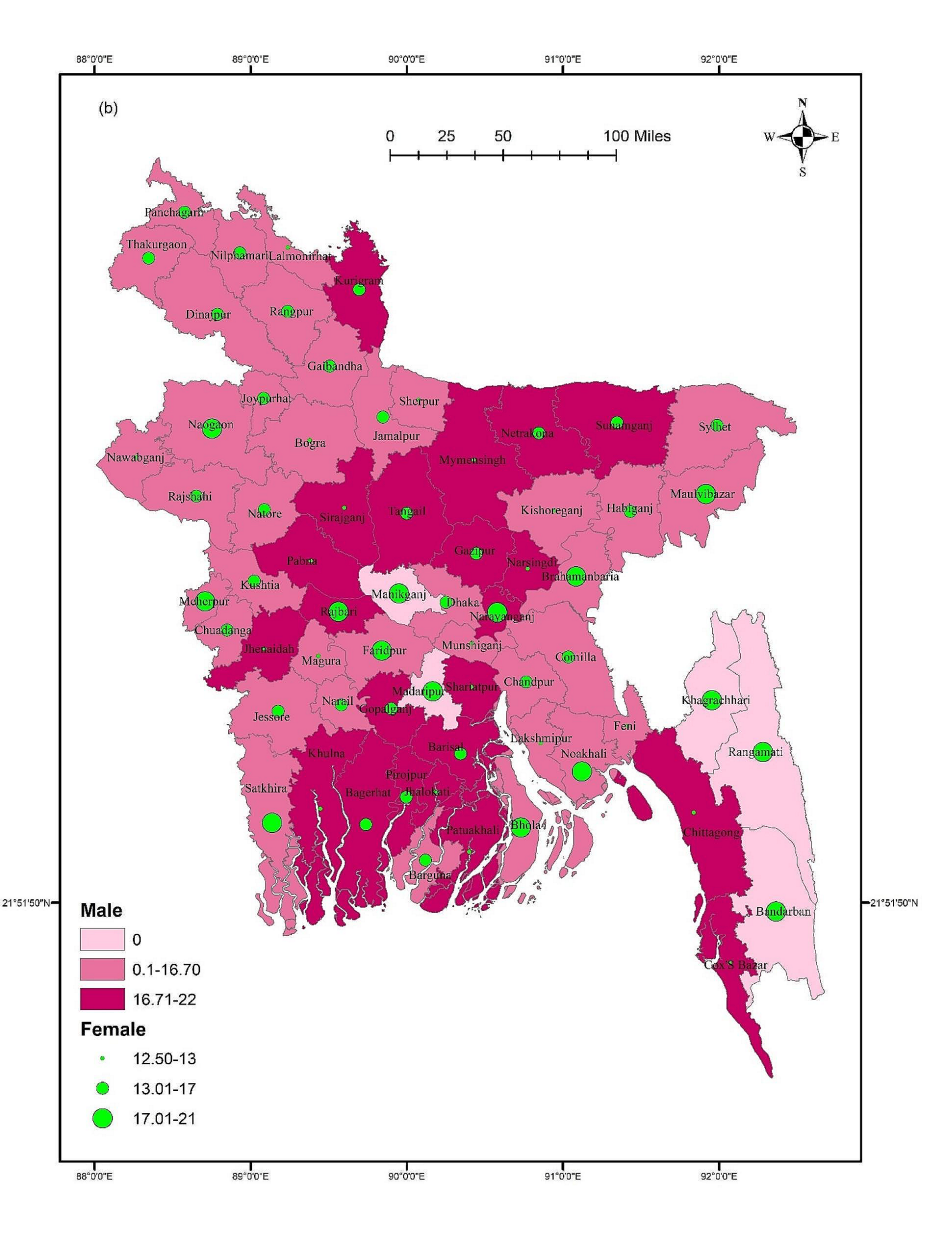
Gender-based mean score of digital addiction across districts

**Fig. 4:**
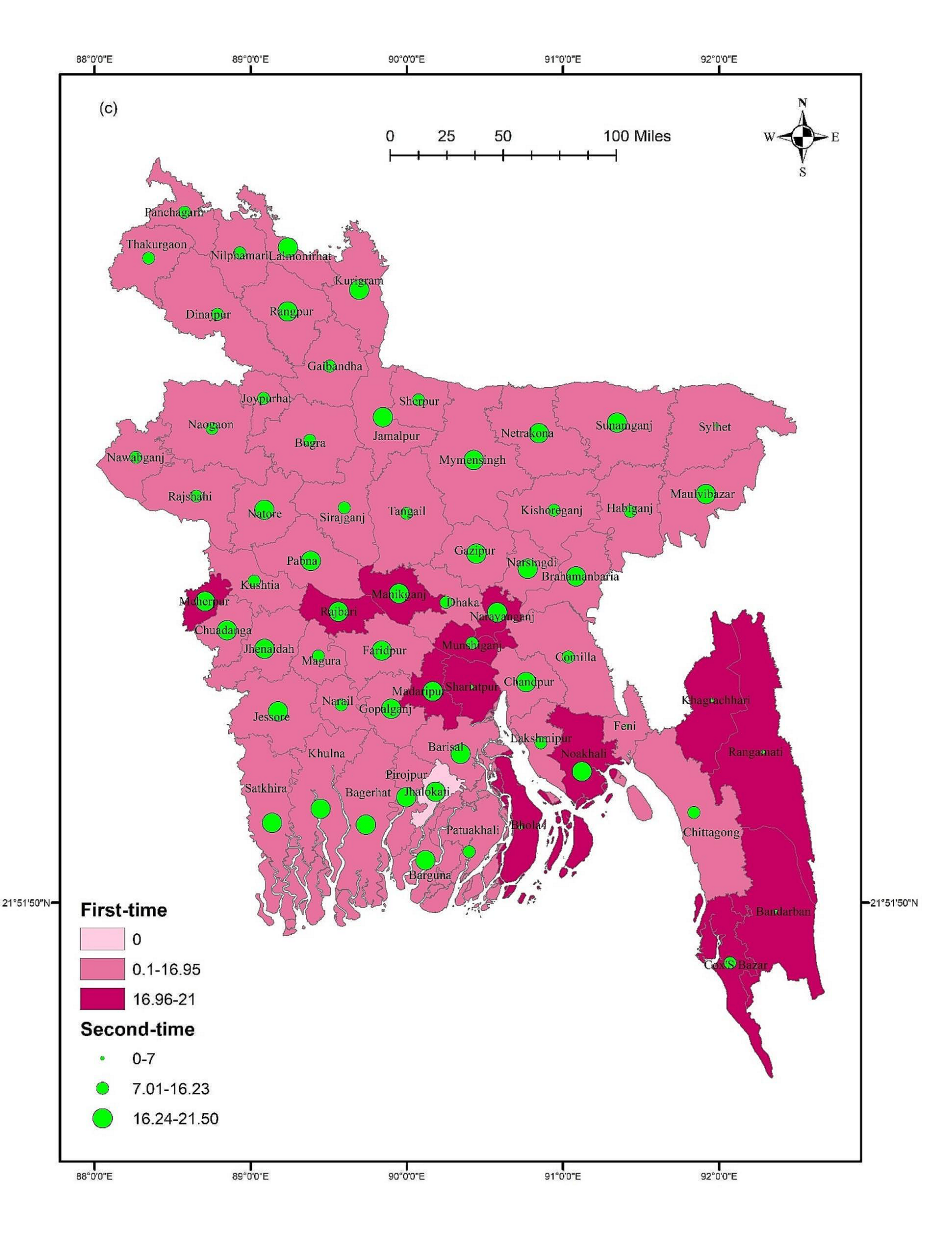
Student status based mean score of digital addiction across districts

The GIS results showed that digital addiction was not significantly distributed across districts (F = 0.729, *p* = 0.946). The mean score of digital addiction was higher in Manikganj, Rajbari, Shariatpur, and Chittagong Hill Tract areas such as Rangamati and Bandarban compared to other districts (Fig. 2). However, for males, some southern districts including Gopalganj, Barisal, Khulna, Pirojpur, Bagerhat, and Patuakhali have higher mean scores of digital addictions. In terms of females, Naogaon, Naraynganj, Nokhali, Bhola, and Chittagong Hill Tract areas such as Khagrachari, Rangamati, and Bandarban showed higher mean scores. Though, gender did not show any significant difference across districts (Male: F = 1.118, *p* = 0.259; Female: F = 0.711, *p* = 0.957) (Fig. 3). Student status did not show any significant differences across districts (first-time test takers: F = 0.611, *p* = 0.992; and repeat test-takers: F = 0.973, *p* = 0.534). Within the first-time test takers, students belonging to Manikganj, Rajbari, Noakhali, Shariatpur, and Chittagong Hill Tract areas and Bandarban showed higher mean scores for digital addiction. However, Rangpur, Kurigram, Jamalpur, Mymensingh, Gazipur, Sunamganj, and some southern districts including Barisal, Bagerhat, Barguna, Satkhira showed higher mean scores for digital addiction for the repeat test-takers (Fig. 4).

## Discussion

This study is the first to investigate the magnitude of digital addiction, and its associations with related socio-demographic, admission-test related and mental health-related variables among students taking university entrance tests in Bangladesh. Spatial mappings were used to provide insights on nationwide distributions of digital addiction, where *post-hoc* associations of district-based digital addiction with respect to gender and student status were also conducted. However, the results suggested that 33.1% of test-taking students were digitally addicted, with a mean score of 16.06 ± 5.58. Noticeably, repeat test takers exhibited a non-significantly higher prevalence of digital addiction than first-time test takers (42.7% vs. 39.1%). The potential factors predicted for digital addiction were student status, satisfaction with previous mock tests, average monthly expenditure during the admission test preparation, and depression. However, the GIS distribution revealed that digital addiction was higher in the districts of Manikganj, Rajbari, Shariatpur, and Chittagong Hill Tract areas, including Rangamati, and Bandarban.

The overall mean score for digital addiction was 16.06 ± 5.58 (score range: 6–30). Previous studies conducted among Turkish children aged 9–12 years (*n* = 506) reported that the mean score of digital addiction was 51.14 ± 20.88 (out of 125 of the 24-item digital addiction scale for children) [27]. Another study conducted in Turkiye’s Mediterranean region among 297 high school students from four institutes reported that the mean score of digital game addiction was 63.71 ± 20.62 [28]. When to the prevalence of digital addiction, that was 33.1% in the present study, which is slightly lower than a recently conducted Bangladeshi study of 769 children aged 8–14 years reported 46.9% had high gadget addiction [11]. In addition, there is a growing prevalence of digital addiction globally, as estimated by a recent meta-analysis [1]. The global prevalence of the different types of digital addictions ranges between 6.04 and 26.99%, whereas it was 26.99% for smartphone addiction, and 17.42% for social media addiction, 14.22% for internet addiction, 8.23% for cybersex addiction, and 6.04% gaming addiction [1]. In Bangladesh, the prevalence of problematic internet use and related other addictive behaviors differs across studies due to the utilization of different cutoff scores as well as scales (for details, please refer to Griffiths and Mamun [9] and Jahan et al. [5]). Specifically, among the school-going adolescents (*n* = 502), a prevalence of 88.25% was reported in the country [10]; whereas a similar rate of problematic smartphone use (86.9%) was reported among high school, medical college, and university students [7], and 29.1% for problematic Facebook use [22] during the COVID-19 pandemic. However, evidence from a Bangladeshi university sample (*n* = 585) found that more than half of them had a moderate level of nomophobia (56.1%) and 34.5% with severe nomophobia; whereas smartphone addiction was identified as a significant mediator between Facebook addiction and nomophobia [29]. However, it is evident that the prevalence reported herein aligns with prior reports from the country, but it is higher than the global levels. However, direct comparison of the previous studies can be limited because of the different instrument used and their cutoff points, as well as cultural and regional differences that might impact on the findings across the studies. It is recommended that there is a necessity of employing standardized assessment procedures and cutoff scores to enable precise cross-study comparisons.

The present study also found that males had significantly higher scores of digital addictions compared to females. Similar findings have been reported in a meta-analysis of global studies from 64 countries, whereas the prevalence of internet addiction (17.15% vs. 11.60%) and gaming addiction (10.71% vs. 4.19%) was higher among males compared to females [1]. However, it is anticipated that repeat test takers would have more digital addiction compared to first-time test takers. Previous studies reported that students who failed in first-time university entrance tests had a higher rate of mental health problems. For instance, studies conducted among similar students taking university entrance tests reported that repeat test takers had higher rates of depression, anxiety, burnout, and suicidal behaviors, as found in the studies conducted before the pandemic [15], and during the pandemic [17]. It is also reported that mental health problems had significant associations with different types of digital addiction, for instance, smartphone addiction [7, 30, 31], Facebook addiction [22], nomophobia [29], internet addiction [6], and social media addiction [32]. The bilateral relationship between mental health problems with digital addiction underscores the possibility of the repeat test takers having a higher chance of digital addiction, but this study did not observe a statistically significant difference.

The present study provides a comprehensive GIS-based distribution to demonstrate the mean scores of digital addictions across districts within the country. Notably, there exist regional differences, with districts such as Manikganj, Rajbari, Shariatpur, and the Chittagong Hill Tract areas exhibiting elevated trends in digital addiction. Though gender-based difference was observed in certain districts, these lack statistical significance. This suggests that both males and females share a similar vulnerability to digital addiction across the studied regions. The present study also reported that there was no significant difference considering student’s status in terms of digital addiction across districts. However, specific regional patterns, for example, greater scores for repeat test-takers in southern regions and Rangpur, may indicate that the causes of digital addiction may vary for those taking the exam for the first time from those retaking it. The observed variations across districts can be attributed to a range of factors, including localized socio-cultural influences, varying levels of technological accessibility, limited access to alternative recreational or educational resources, psychosocial stressors linked to rapid urbanization, peer influence, and other contextual factors. Additional qualitative studies could shed light on these contextual factors, fostering a more comprehensive understanding and paving the way for focused preventive measures among university entrance test takers.

### Recommendations for policy, practice, and parents

The discussion in this paper sheds light on the pressing issue of digital addiction among students taking university entrance tests in Bangladesh, drawing upon current and localized evidence. To effectively address this issue, collaborative efforts at policy, practice, and parental levels are paramount. By implementing these multifaceted recommendations, stakeholders can collectively work towards mitigating the adverse effects of digital addiction and nurturing a healthier digital ecosystem for students in Bangladesh.

- **Reconsideration of Entrance Test Processes**: Given the profound mental health challenges observed among test-taking students, particularly in the context of stressful entrance tests [12], there is a compelling need to reassess and explore alternative processes for entering higher education. A more holistic and supportive approach to this critical juncture is warranted.
- **Integration of Digital Literacy Programs**: Incorporating digital literacy programs into the national curriculum emerges as a crucial step. These programs would empower students with the knowledge and skills necessary for the responsible and balanced use of digital devices and gadgets.
- **Allocation of Resources for Mental Health Support**: Educational institutions should allocate resources for the development of mental health support services. These services can effectively address issues such as depression and anxiety, which are intricately linked to digital addiction.
- **Integration of Digital Wellness Programs**: Educational institutions are encouraged to integrate digital wellness programs into their support services. This initiative would involve offering counseling and resources to students grappling with digital addiction, thereby promoting a healthier digital environment.
- **Promotion of Alternative Activities**: Advocacy for alternative activities, including sports, arts, and community engagement, is pivotal. This strategy aims to reduce excessive screen time and foster a more balanced lifestyle among students.
- **Parental Awareness and Involvement**: Acknowledging the pivotal role of parents, concerted efforts should be made to enhance parental awareness regarding digital media consumption. Workshops and seminars, organized by both educational and social institutions, can effectively address the significance of a healthy digital life and the importance of parental mediation.
- **Setting Boundaries and Encouraging Balanced Lifestyles**: Parents are encouraged to set boundaries and limits on the use of digital devices, especially during critical periods such as exam preparation. Promoting engagement in alternative activities, including physical exercise and household chores, contributes to ensuring a balanced lifestyle for students.

### Strengths and limitations of the study

This study’s strength can be noted as its investigation of digital addiction among a specialized group of students with a large sample that never studied inside and/or outside Bangladesh. The application of GIS in the distribution of digital addiction across districts identifies the spatial patterns and risky zones. Additionally, adopting a validated scale to measure digital addiction could create a path for future studies. However, several limitations should be acknowledged in this study. Firstly, the cross-sectional nature of the study limits the establishment of causal relationships between variables. Future research employing longitudinal designs could offer more robust evidence in this regard. Secondly, the sampling distribution is biased toward first-time test-takers. While a post hoc analysis addresses the bias, caution is warranted in generalizing the findings. Moreover, self-reporting survey methods, introduce the possibilities of social desirability bias and memory recall bias. To enhance data reliability and minimize biases, future research should consider adopting diverse and representative sampling methods. Furthermore, recognizing the focus on university entrance test-takers underscores the need for caution in extending findings to broader student populations, and variations in individual perceptions may contribute to response biases, adding complexity to result interpretation. Lastly, future studies are recommended to explore specific addiction measures, such as Facebook, Smartphones, YouTube, Instagram, Online gaming, and so on.

## Conclusions

The present study found that approximately one-third of the participants had digital device addiction. Notably, repeat test-takers, those who were not satisfied with their previous mock tests, high monthly expenditures during admission test preparations, and those suffering from depression were at higher risk of problematic use of digital devices. The findings emphasize the pressing need for comprehensive measures to tackle the rising prevalence of digital addiction among students who are going to pursue their university education in Bangladesh. The recommendations encompass revisiting entrance test processes, integrating digital literacy programs, allocating resources for mental health support, and promoting digital wellness programs. Besides, encouraging alternative activities, enhancing parental awareness, and setting boundaries contribute to nurturing a healthier digital environment for students in Bangladesh.

### Electronic supplementary material

Below is the link to the electronic supplementary material.

Supplementary Material 1

## Data Availability

The datasets will be made available to appropriate academic parties upon request from the corresponding author.
