# Supplementary material for: Prevalence and factors associated with digital addiction among students taking university entrance tests: a GIS-based study
Source: BMC Psychiatry. 2024 Apr 25;24:322. doi: 10.1186/s12888-024-05737-9 (PMC11044373; doi:10.1186/s12888-024-05737-9)
Supplement: Supplementary file 1 — Supplementary Material 1 [file 12888_2024_5737_MOESM1_ESM.pdf]

Appendix

Digital Addiction Scale

1. Spent a lot of time thinking about *digital devices and/or gadgets* or planned use of digital devices and/or gadgets?

2. Felt and urge to use *digital devices and/or gadgets* more and more?

3. Used *digital devices and/or gadgets* in order to forget about personal problems?

4. Tried to cut down the use of *digital devices and/or gadgets* without success?

5. Become restless or troubled if you have been prohibited from using *digital devices and/or gadgets*?

6. Used *digital devices and/or gadgets* so much that it has had a negative impact on your job/studies?

Bangla Digital Addiction Scale

**আপনি যদি নিচের মতামতগুলোর সাথে কিভাবে একমত সেটাতে (✔) টিক দিন**	খুব কম	কম	মাঝেমধ্যে	প্রায়সময়	সবসময়
১) প্রায়ই আপনি কি অনেক সময় নষ্ট করেন এটা ভেবে যে কিভাবে ডিজিটাল ডিভাইস ব্যবহার করবেন?					
২) বারবার ডিজিটাল ডিভাইস ব্যবহার করার জন্য আপনার মনের মধ্যে কি এক প্রকার উদ্দীপনা তৈরি হয়?					
৩) সাধারণত নিজের সমস্যা ভুলে থাকার জন্যে আপনি কি ডিজিটাল ডিভাইস ব্যবহার করেন?					
৪) অনেকবার চেষ্টা করার পরেও আপনি কি মাত্রাতিরিক্ত ডিজিটাল ডিভাইস ব্যবহার কমাতে পারেন নি?					
৫) ডিজিটাল ডিভাইস থেকে দীর্ঘ সময় দূরে থাকলে আপনার কি অস্থির ও উদ্বিগ্ন লাগে?					
৬) ডিজিটাল ডিভাইস এত বেশি ব্যবহার করছেন যে, এর কারণে আপনার পড়াশোনা অনেকটাই ব্যাহত হচ্ছে?					
